# Splicing Factor Transcript Abundance in Saliva as a Diagnostic Tool for Breast Cancer

**DOI:** 10.3390/genes11080880

**Published:** 2020-08-03

**Authors:** Mercedes Bentata, Guy Morgenstern, Yuval Nevo, Gillian Kay, Avital Granit Mizrahi, Mark Temper, Ofra Maimon, Liza Monas, Reham Basheer, Asa Ben-Hur, Tamar Peretz, Maayan Salton

**Affiliations:** 1 Department of Biochemistry and Molecular Biology, The Institute for Medical Research Israel–Canada, Faculty of Medicine, The Hebrew University of Jerusalem, Jerusalem 9112102, Israel; mercedes.bentata@mail.huji.ac.il (M.B.); morgenstern.guy@gmail.com (G.M.); gilliank@ekmd.huji.ac.il (G.K.); 2 Info-CORE, Bioinformatics Unit of the I-CORE at the Hebrew University of Jerusalem and Hadassah Medical Center, Jerusalem 9112102, Israel; yuval.nevo@mail.huji.ac.il; 3 Sharett Institute of Oncology, Hadassah-Hebrew University Medical Center, Hebrew University Medical School, Jerusalem 9112102, Israel; granit@hadassah.org.il (A.G.M.); markt@hadassah.org.il (M.T.); ofra406@hadassah.org.il (O.M.); lizam@hadassah.org.il (L.M.); basir@hadassah.org.il (R.B.); tamary@hadassah.org.il (T.P.); 4 Department of Computer Science, Colorado State University, Fort Collins, CO 80523, USA; asa@cs.colostate.edu

**Keywords:** splicing factors, saliva, diagnosis, breast cancer

## Abstract

Breast cancer is the second leading cause of death in women above 60 years in the US. Screening mammography is recommended for women above 50 years; however, 22% of breast cancer cases are diagnosed in women below this age. We set out to develop a test based on the detection of cell-free RNA from saliva. To this end, we sequenced RNA from a pool of ten women. The 1254 transcripts identified were enriched for genes with an annotation of alternative pre-mRNA splicing. Pre-mRNA splicing is a tightly regulated process and its misregulation in cancer cells promotes the formation of cancer-driving isoforms. For these reasons, we chose to focus on splicing factors as biomarkers for the early detection of breast cancer. We found that the level of the splicing factors is unique to each woman and consistent in the same woman at different time points. Next, we extracted RNA from 36 healthy subjects and 31 breast cancer patients. Recording the mRNA level of seven splicing factors in these samples demonstrated that the combination of all these factors is different in the two groups (*p* value = 0.005). Our results demonstrate a differential abundance of splicing factor mRNA in the saliva of breast cancer patients.

## 1. Introduction

An early diagnosis is crucial to improve the morbidity and mortality of breast cancer. More than 90% of women diagnosed with breast cancer at the earliest stage survive the disease for at least five years, compared to a survival rate of around 15% of women diagnosed at the most advanced stage of disease [1]. Two million women were diagnosed with breast cancer worldwide in 2018 [2]; of which approximately 7% were diagnosed before the age of 40 and 22% before the age of 50 [3]. This disease accounts for more than 40% of all cancers in women before the age of 40 [4]. Survival rates are worse in younger women, and a multivariate analysis has shown a younger age to be an independent predictor of an adverse outcome [4]. In most of the developed world, screening for the general population using mammography begins at the age of 50 in accordance with the World Health Organization’s recommendations [5]. Thus, it is clear that a method for early screening—from the age of 20—would greatly benefit the female population.

To develop a non-invasive diagnostic test, we decided to search for breast cancer biomarkers in saliva. Saliva is a unique fluid, and interest in it as a diagnostic medium has increased over the last decade. Saliva harbors a wide spectrum of nucleic acids, proteins/peptides, electrolytes, and hormones that are derived from local and systemic origins. The most common route for substances to migrate from blood to saliva is via unaided or passive diffusion. Salivary mRNA, protein, metabolites and carbohydrates have been shown to be breast cancer biomarkers [6]. The c-erbB-2 protein and soluble HER-2 receptor in saliva were found to be elevated in breast cancer patients relative to healthy women [7,8,9]. In addition, a combined detection of eight mRNA biomarkers and one protein was found to serve as a good diagnostic tool for breast cancer [10]. Furthermore, a large spectrum of metabolites were differentially detected in breast cancer patients [11,12].

The biggest challenge in using saliva as a diagnostic tool is that the analytes are at concentrations that are around a thousandth of those in the blood [13]. For this reason, we focused on the RNA in saliva as RNA detection systems, such as real-time PCR, can amplify very small amounts of RNA. In addition, the detection of RNA is possible in clinical settings and it was recently discovered that RNA reaches the saliva from all parts of the body via exosomes [14]. Exosomes are small cell-secreted vesicles of about 30–100 nm, derived by pinching off the plasma membrane. Exosomes retain their cytoplasmic contents and thus transport proteins, mRNAs and microRNAs distinctive to their cell of origin. Exosomes function as versatile promoters in the tumorigenesis, metastasis and development of drug resistance in breast cancer. In addition, breast cancer exosomes have been shown to increase proliferation and reduce the apoptosis of the surrounding normal breast cells [15]. Furthermore, exosomes that carry RNA to saliva have been shown to be able to migrate from mice lung tumors [16].

We started our investigation by sequencing cell-free saliva RNA from ten healthy women to reveal the full mRNA transcriptome. We discovered an enrichment of genes with a functional annotation in alternative splicing. We focused on seven splicing factors and found that those were consistent in the same woman on different days. Furthermore, these splicing factors had differential abundances when samples from 36 healthy subjects and 31 breast cancer patients were compared. Our results demonstrate that mRNA’s abundance of splicing factors in saliva can serve as an indicator for breast cancer.

## 2. Materials and Methods

### 2.1. Ethics Statement

Human saliva samples were obtained from healthy women and estrogen receptor-positive (ER+) breast cancer patients under the Hadassah Institutional Helsinki committee, approval no.“0.346-12-HMO”. All patients gave written informed consent.

### 2.2. RNA Isolation and Real-Time PCR

Saliva was kept at −20 °C before extraction. Saliva was centrifuged at 4 °C for 15 min at 2600 g. RNA was extracted from 400 μL of the supernatant using the NucleoSpin RNA XS Kit (Macherey-Nagel, Dueren, Germany) according to the manufacturer’s instructions, with elution in 30 μL. cDNA synthesis was carried out with 23 μL of RNA using the Quanta cDNA Reverse Transcription Kit (QuantaBio, Beverly, MA, USA) according to the manufacturer’s instructions. Real-time PCR was performed with the iTaq Supermix (BioRad Rishon Le Zion, Israel) on the Bio-Rad iCycler. The comparative Ct method was used to quantify transcripts, and ∆Ct was measured in triplicate. The primers used in this study are provided in Supplementary Table S1.

### 2.3. RNA-Seq

The library was prepared using the KAPA stranded mRNA-seq library kit (Roche KK-KK8421) with a modification to the protocol—the RNA library preparation was initiated from the fragmentation step, after which the regular protocol was followed. The library quality control was done using Qubit 4 (Invitrogen, Loughborough, UK) and TapeStation 2200 (Agilent, Waldbronn, Germany). The library was diluted to 4 nM and sequenced on NextSeq 500, using the NextSeq high output kit v2, 75 cycles (Illumina, San Diego, CA, USA).

### 2.4. RNA-Seq Analysis

Trimming and filtering of raw reads: The raw reads (fastq files) were inspected for quality issues with FastQC (v0.11.5, http://www.bioinformatics.babraham.ac.uk/projects/fastqc/). According to the FastQC report, reads were quality-trimmed at both ends, then poly-G sequences (NextSeq’s no signal) were removed from the 3′ end, then adapter sequences were removed from the 3′ end, and finally low-quality reads were filtered out. Quality-trimming was done using in-house Perl scripts, with a quality threshold of 32. In short, the scripts use a sliding window of 5 bases from the read’s end and trim one base at a time until the average quality of the window passes the given threshold. The poly-G and adapter sequences were removed with cutadapt (version 1.12, http://cutadapt.readthedocs.org/en/stable/) [17], using a minimal overlap of 1 (-O parameter), allowing for read wildcards, and filtering out reads that became shorter than 15 nt (-m parameter). Filtering the remaining reads was done with the FastQ_quality_filter program of the FASTX package (version 0.0.14, http://hannonlab.cshl.edu/fastx_toolkit/), with a quality threshold of 20 at 80% or more of the reads’ positions. Mapping and differential expression: The processed FastQ files were mapped to the human transcriptome and genome using TopHat (v2.1.1) [18]. The genome version was GRCh38, with annotations from Ensembl release 89. Mapping allowed up to 10 mismatches per read, a maximum gap of 5 bases, and a total edit distance of 15 (full command: tophat-G genes.gtf-N 10–read-gap-length 5–read-edit-dist 15–segment-length 20–read-realign-edit-dist 3–no-coverage-search–library-type fr-firststrand genome processed.fastq).

Quantification was done using HTSeq-count (version 0.6.0, http://www-huber.embl.de/users/anders/HTSeq/doc/count.html) [19]. Strand information was set to “reverse”, and an annotation file that lacked information for genes of type IG, TR, artifact, miRNA, Mt_rRNA, Mt_tRNA, ncRNA, piRNA, pre-miRNA, rRNA, ribozyme, sRNA, scRNA, scaRNA, siRNA, snRNA, snoRNA, tRNA and vaultRNA was used.

Genes with a sum of counts less than three over all the samples were filtered out. Finally, results were combined with the gene details (such as symbol, Entrez accession, etc.), taken from the results of a BioMart query (Ensembl, release 89), to produce the final Excel file.

### 2.5. Data Availability

The RNA-seq data have been deposited to the Gene Expression Omnibus (GEO) with the dataset identifier GSE145796.

## 3. Results

### 3.1. Detection of Splicing Factors mRNA in Cell-Free Saliva

We first conducted RNA-seq to reveal the full transcriptome of cell-free saliva. Since we wanted a general picture of the RNA species and not something that is specific to one woman, we pooled saliva cell-free RNA from ten different women. Pooling ten samples also helped us overcome the problem of the small quantity of RNA (<1 ng) extracted from each sample.

**Figure 1:**
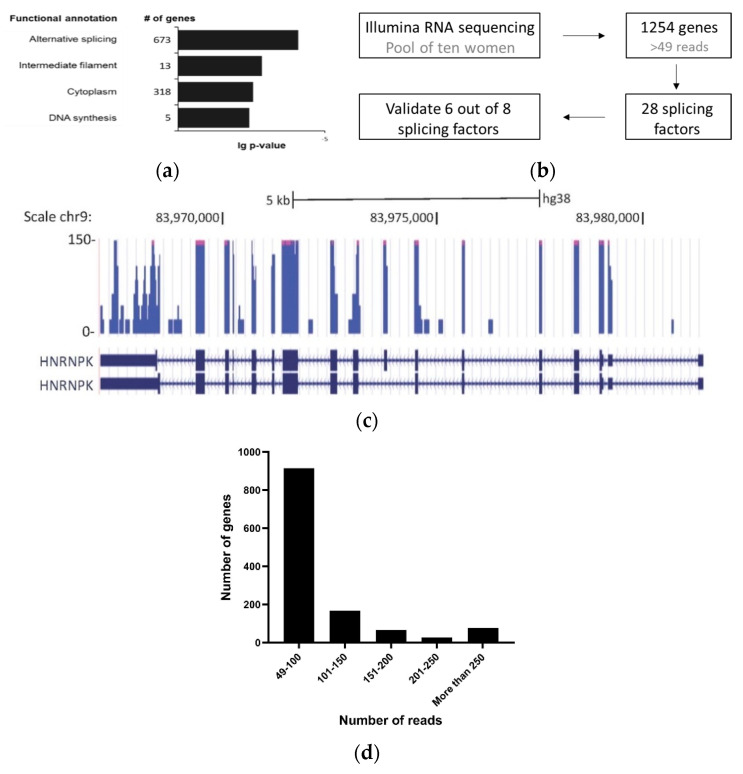
Enrichment of alternative splicing proteins in saliva cell-free RNA. The RNA-seq of cell-free RNA was extracted from the saliva of ten women. (**a**) Functional analysis of the 1254 transcripts identified was conducted using the DAVID functional annotation tool (DAVID, https://david.ncifcrf.gov/). (**b**) Schematic representation of the splicing factors chosen from the RNA-seq to use for the diagnostic test. (**c**) UCSC Genome Browser screen shot of RNA-seq data at the HNRNPK locus gene (reference genomeGRCh38/hg38). HNRNPK exon 8 alternative splicing was indicated by the RNA-seq results. (**d**) Transcript abundance in cell-free saliva represented by the number of genes relative to number of reads.

We set the threshold of 49 reads per transcript as an indicator for a reasonable presence of the transcript in saliva. A total of 1254 transcripts were thus identified; using the DAVID function annotation tool (DAVID, https://david.ncifcrf.gov/) [20,21], we found that this list of genes is enriched for genes with a functional annotation of alternative splicing, intermediate filament, cytoplasm and DNA synthesis (Figure 1a). The genes with a functional annotation of alternative splicing accounted for 673 genes. Out of 1254 transcripts, we identified 28 splicing factors, amounting to around 40% of the 71 known human splicing factors described in SpliceAid-F [22] (Supplementary Table S2). This result was of great interest since alternative splicing is known to drive cancer [23,24,25,26].

The process of pre-mRNA splicing removes introns that are part of most human genes [27]. The splicing reaction is catalyzed by the spliceosome, a multi-subunit complex comprised of small noncoding RNAs (U1, U2, U4, U5 and U6) and a myriad of associated proteins [28]. While many exons are constitutively spliced together, alternative splicing is a process during which specific exons are selectively included or excluded [28]. Alternative splicing is of great physiological relevance since combinatorial control mechanisms regulate alternative exon recognition, which enables splicing programs to coordinate the generation of many mRNA isoforms from single genes. The various protein isoforms produced can have different functions and, as such, alternative splicing contributes significantly to the regulation of cellular functions [29].

Alterations in splicing behavior in cancer may be caused by changes in the expression of splicing factors that can dictate an oncogenic splicing pattern [30] or by mutations that give rise to a specific splicing isoform that can promote cancer [31]. Specifically, in breast cancer, changes in alternative splicing driven by abnormal expressions of splicing factors have been reported [25,32].

### 3.2. Markers in Saliva Are Consistent in the Same Woman on Different Days

In light of our finding, we measured the amount of the splicing factors in saliva using real-time PCR. We designed primers for eight splicing factors and validated them in cell-free RNA extracted from the saliva samples of three healthy women. Six out of the eight splicing factors were successfully identified (Figure 1b, Supplementary Figure S1a). The six splicing factors are: HNRNPA1, HNRNPA2B1, HNRNPA3, HNRNPK, PTBP1 and SRSF6. We also added primers for an isoform of HNRNPK with exon 8 inclusion which was sequenced in saliva (Figure 1c and Supplementary Figure S1b–g). Five out of the six splicing factors have been shown before to be overexpressed in breast cancer: HNRNPA1 [33], HNRNPA2B1 [34,35], HNRNPK [36,37], PTBP1 [38,39] and SRSF6 [40,41,42]. For normalization we chose PPIA, which is routinely used as an endogenous control in real-time experiments [43] and we have found it to be highly abundant relative to the other genes sequenced, with a number of 197 reads in cell-free RNA from saliva (Figure 1d). For this reason, we used it here as our normalizer to measure the relative abundance of the splicing factors.

**Figure 2:**
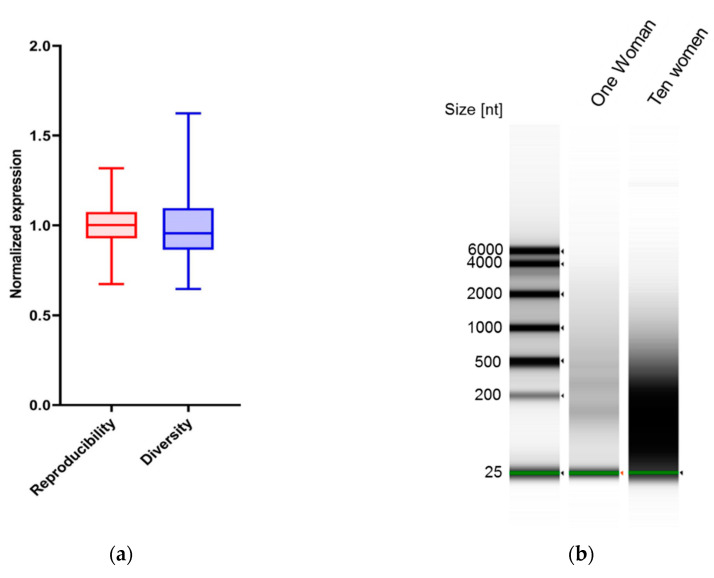
Reproducibility and diversity of splicing factor transcripts in cell-free saliva. For the reproducibility measurement, RNA was extracted from cell-free saliva from the same woman on different days. Diversity was measured by the extraction of RNA from ten different menstruating women with ages ranging from 20 y to 50 y (**a**) Real-time PCR was conducted for HNRNPA1, HNRNPA2B1, HNRNPA3, HNRNPK, HNRNPK exon 8 inclusion, PTBP1 and SRSF6 and normalized to PPIA. Relative expression levels were summed and plotted. (**b**) RNA from diversity and reproducibility groups was pooled and visualized on an Agilent bioanalyzer.

We checked next the robustness of the RNA identification in cell-free saliva. We extracted saliva RNA from the same woman on ten different days and compared the amount and identity of specific splicing factors on each day (Figure 2a). We used a coefficient of variation (CV) statistical analysis to evaluate the reproducibility of the method and RNA identification (Supplementary Figure S2). Our results demonstrate a strong reproducibility in the ten samples. Taken together, this result shows that the RNA identification is robust and consistent. To check the feasibility of using RNA identification as a diagnostic tool, we wanted to make sure that there are different amounts of the splicing factors in different women. We extracted cell-free saliva RNA from ten different women and quantified our seven targets (Figure 2a). Our statistical analysis concluded that the variance between women is significantly greater than that of the same woman on different days. Similarly, visualizing the RNA using an Agilent bioanalyzer demonstrated more variance in the group of ten women (Figure 2b). These results serve as a proof-of-concept that cell-free saliva mRNA can be used as a “fingerprint” for a specific woman.

### 3.3. Transcript Level of Splicing Factors Can Be Used as Markers for Breast Cancer in Cell-Free Saliva

Next, we conducted a comparative study to determine the differential splicing factor abundance of healthy women compared to that of women diagnosed with estrogen receptor-positive (ER+) breast cancer. We collected 36 saliva samples from healthy subjects visiting a high-risk clinic at the Sharett Institute of Oncology at the Hadassah Medical Center. The women visiting the clinic had a family history of breast or ovarian cancer, and in some the cancer is a result of mutations in breast cancer predisposing genes (such as BRCA1/2). Saliva from 31 ER+ breast cancer patients was obtained from the Oncology ambulatory services unit at the Sharett Institute. The specific details of the disease, as well as the treatment, can be found in Table 1. We concentrated on a homogenous group of women with breast cancer, and focused on women with ER positive diseases only, excluding women with triple negative diseases. 

**Figure 3:**
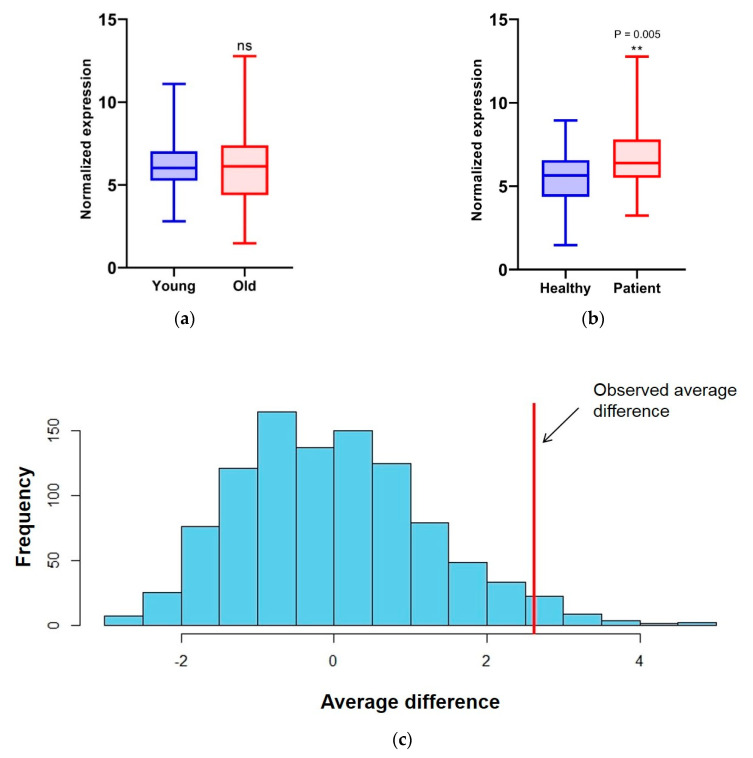
Splicing factors are differentially abundant in healthy and breast cancer patients (**a**,**b**). RNA was extracted from the cell-free saliva of 36 healthy and 31 breast cancer patients. Real-time PCR was conducted for HNRNPA1, HNRNPA2B1, HNRNPA3, HNRNPK, HNRNPK exon 8 inclusion, PTBP1 and SRSF6 and normalized to PPIA. Relative expression levels were summed and plotted. Thirty-six healthy and 31 breast cancer patients were pooled together and divided based on age—to “young” and “old” groups. Relative expression levels were summed and plotted (**a**). Healthy and 31 breast cancer groups were plotted (**b**). (**c**) Splicing factor abundance in a sub-group of patients treated with avastin (*n* = 3) were compared to the remaining group of patients by permutation testing (1000 times).

We extracted RNA from all the saliva samples and conducted RT-qPCR for the seven targets chosen in addition to PPIA, the gene we chose as a normalizer. We calculated the amount of each splicing factor and found that the HNRNPA2B1 level was slightly lower in breast cancer patients and all other splicing factor levels higher; this difference reached significance for PTBP1 (*p* < 0.05), HNRNPK_ex89 (*p* < 0.05) and SRSF6 (*p* < 0.01, Supplementary Figure S3a). To simplify our calculations, we calculated the sum of all the changes (ΣΔ) in the splicing factor levels. Since the average age of the two groups was significantly different (healthy: 40 ± 12, patients: 53 ± 13), we divided all the healthy and breast cancer patient samples into a “young” and “old” group based on their age at saliva collection. Our results showed no significant difference in the total splicing factor abundance (Figure 3a) between the young and old groups. Interestingly, when we divided the healthy subjects group and the patients group each into the “young” and “old” groups, we only found a significant difference in splicing factor abundance between the “young” and “old” subjects in the healthy group (Supplementary Figure S3b–e). Comparing the healthy and breast cancer patient groups, our results clearly show a significant difference between the two groups (*p* value = 0.005, Figure 3b).

Following the collection of the data, we explored what factors could affect the abundance of the splicing factors and evaluated the different treatment characteristics. To this end, we ran a permutation test in which a random sub-group of patients was chosen and compared to the remaining patients population (1000 times). The only significant difference in the splicing factor abundance associated with treatment was with the anti-VEGFR treatment, avastin (*n* = 3; *p* value = 0.033, Figure 3c).

## 4. Discussion

Pre-mRNA splicing regulators are emerging as a new class of oncoproteins or tumor suppressors [44]. Interestingly, splicing factors in solid tumors often display copy-number variation or changes in expression levels [45]. A change in the expression of splicing factors will lead to dis-regulation of alternative splicing. Altering alternative splicing can promote cancer by regulating oncogenic and tumor-suppressor isoforms. An RNA-seq of tumors from the three most common types of breast tumors (TNBC, non-TNBC and HER2-positive) demonstrate an altered alternative splicing relative to normal tissue [46]. The function of the differential isoforms was found to be related to cell progression and metastasis [46]. The significant changes in the alternative splicing in tumors led to the use of isoform ratios as markers for breast cancer [32]. It was shown that using 12 such isoforms as biomarkers can distinguish a breast cancer tumor from normal tissue and moreover specify the grade level of the tumor [32]. Many splicing factors have been shown to have a role in breast cancer and to promote its progression, including SRSF1 [30,47,48], SRSF3 [49], SRSF5 [50], SRSF6 [40,41,42], SRSF10 [51], HNRNPA1 [33], HNRNPA2B1 [34,35,52], HNRNPM [53], HNRNPK [36,37], HNRNPL [54], RBFOX2 [55], ESRP1/2 [55], PTBP1 [38,39] RBM5/10 [56], Sam68 [57] and FOX2 [58]. Since our sequencing of saliva RNA identified 28 splicing factors out of the known 71, we focused on this group as a possible marker.

The difference in the expression level of this group of splicing factors between healthy and breast cancer patients (*p* value = 0.005) led us to speculate that splicing factors are indeed strong markers for breast cancer. Although this difference is significant when the healthy and breast cancer patient groups are compared, a single patient sample cannot be distinguished from the group of healthy subjects. We predict that increasing the number of splicing factors examined could strengthen our prediction dramatically. It is technically challenging to measure many targets in real-time PCR since the amount of RNA we extract from cell-free saliva is very small (<1 ng). Since most of the splicing factors we chose are abundant in saliva, we expect that doubling the number of targets could give us a robust result. In addition, increasing the sample size of healthy and breast cancer patients should also improve our results significantly. Having said that, our results are far weaker than quantifying splicing factors in the tumor itself, as discussed above [32]. This is of course a result of the distance of the saliva from the tumor and the low amount of RNA in saliva. Results from a study on GFP tumors in mice found that the GFP RNA levels are similar in saliva and blood [13]. For this reason, we predict that tumor RNA will give a better diagnosis compared to both saliva and blood.

The main diagnostic test for breast cancer is mammography, but screening for the general population in the US starts at the age of 40 [59], while approximately 7% of women with breast cancer are diagnosed before that age. In addition, in this age group mammography has a false negative rate of about 30–40% due to hormonal activity, and adding saliva testing can contribute to the early detection of breast cancer [60]. Breast cancer accounts for more than 40% of all cancer in women in this age group. The survival rates are worse than those in older women, and a multivariate analysis has shown younger age to be an independent predictor of adverse outcomes [4]. For this reason, we were interested in developing a non-invasive diagnostic test for this type of cancer that will provide an indication for a secondary test. Since saliva can serve as an indicator of many systemic diseases, and the splicing factor abundance is known to change in many types of cancer, we predict that measuring splicing factors could serve as a diagnostic tool for several types of cancer.

The RNA-seq data in this study show that alternative splicing-related genes are highly present in saliva cell-free RNA. This result is interesting and might suggest that splicing-related genes are stable or enriched in exosomes. Our results so far do not indicate the cause of this enrichment. In this study, we mainly focused on splicing factors, which are only one part of the immense field of splicing regulation. Thus, we suggest future studies look into the other aspects related to this process, such as the target genes of the splicing factors and their different isoforms. RNA-seq and other high throughput and bioinformatic techniques will enable a general view of the splicing landscape of RNA present in saliva to be obtained, as well as changes in the specific isoforms. Next, it will be essential to understand the biological meaning of these changes and whether they are also related to the health status of the woman (healthy vs. breast cancer patient), and thus could be used as biomarkers for the tumor itself.

In addition, the experimental group of our study consisted of breast cancer patients in very late stages of the disease, with more than 70% of the patients having developed metastases. We thought that studying this subpopulation first would answer the question of whether there are differences in the saliva free-cell RNA profile of patients. For future studies, the next step will be investigating whether these differences are also found at earlier stages of the disease and even if they are found in women who will later go on to develop tumors. If so, this study assures that saliva is a biofluid that can be used to detect changes emerging from a tumor development process even at the earliest stages.

## Figures and Tables

**Table 1:** Details of patients’ characteristics (including disease and treatment).

#	Age (Years) at:			IDC ^1^	ILC ^2^	HER2	Site of Metastasis	Treatment
	Collection	Diagnosis	Death					
1	66	43	67	√	√	+	Liver, lung, bone	Chemotherapy (carboplatin/gemzar)
2	57	56	58	√	×	−	Liver, bone	Hormonal and Palbocyclib
3	36	27	37	√	×	−	Bone, lung	Chemotherapy (cyclophosphamide/methotrexate/5FU ^3^)
4	55	54		√	×	+	Local disease	Chemotherapy (adriamycin/cyclophosphamide (neoadjuvant))
5	53	49	55	√	×	−	Bone, liver	Avastin
6	40	33	41	√	×	−	Bone, liver	Avastin and Chemotherapy (cisplatin/gemzar)
7	33	33		√	×	+	Local disease	Targeted therapy for HER2 and Chemotherapy (navalbine (neaoadjuvant))
8	64	54	66	√	×	−	Bone, liver	Chemotherapy (carboplatin/gemzar)
9	38	32	39	√	×	+	Bone, lung	Targeted therapy for HER2
10	37	36		√	×	+	Brain	Targeted therapy for HER2
11	62	46	64	√	×	−	Liver, bone	Chemotherapy (carboplatin/gemzar)
12	84	76		√	×	+	Local disease	Targeted therapy for HER2 and Chemotherapy (navalbine (neaoadjuvant))
13	54	36	56	√	×	−	Bone, lung, liver	Chemotherapy (cisplatin/leucovorin/5FU ^3^)
14	58	58		√	×	+		Targeted therapy for HER2
15	71	69	72	√	×	−	Lung, liver, bone	Hormonal
16	53	53		√	×	−		Chemotherapy (taxol (adjuvant))
17	55	49		√	×	−	Lung, bone	Avastin and Chemotherapy (navalbine)
18	85	80	85	√	×	−	Liver, bone, lung	Chemotherapy (carboplatin)
19	49	48		√	×	+		Targeted therapy for HER2
20	35	35		√	×	−	Local disease	Chemotherapy (taxol (neoadjuvant))
21	67	64	68	×	√	−	Brain, bone, lung	Chemotherapy (carboplatin + taxol)
22	47	42	47	√	×	−	Bone, liver	Chemotherapy (carboplatin/gemzar)
23	46	45		√	×	+		Targeted therapy for HER2
24	54	41	55	√	×	−	Bone	Hormonal and Palbocyclib
25	51	51	52	√	×	−	Bone	Hormonal and Palbocyclib
26	35	29	36	√	×	−	Liver, bone, adrenal gland	Hormonal and Afinitor
27	62	62		×	√	−		Chemotherapy (taxol (adjuvant))
28	39	39	40	√	×	−	Bone	Hormonal and Palbocyclib
29	58	52	59	√	×	−	Bone	Chemotherapy (taxol)
30	56	43	57	√	×	−	Liver, bone	Hormonal and Afinitor
31	52	52	53	√	×	−	Bone, liver	Chemotherapy (xeloda)

^1^ IDC: Invasive/infiltrating ductal carcinoma; ^2^ ILC: invasive/infiltrating lobular carcinoma; ^3^ 5FU: fluorouracil.

## Supplementary Materials

The following are available online at https://www.mdpi.com/2073-4425/11/8/880/s1; Table S1: List of primers used in this study; Table S2: List of 28 splicing factors identified in RNA-seq; Figure S1: RNA-seq snap shots of validated splicing factors; Figure S2: Reproducibility and diversity measurement of each splicing factor; Figure S3: Results from cell-free saliva of 36 healthy and 31 breast cancer patients.
