# Supplementary material for: Splicing Factor Transcript Abundance in Saliva as a Diagnostic Tool for Breast Cancer
Source: Genes (Basel). 2020 Aug 3;11(8):880. doi: 10.3390/genes11080880 (PMC7463790; doi:10.3390/genes11080880)
Supplement: Supplementary file 1 [file genes-11-00880-s001.pdf]

Supplementary Fig. S1

a

Target	Ct <35 and a single melt peak
HNRNPA1	Yes
HNRNPA2B1	Yes
HNRNPA3	Yes
HNRNPH1	No
HNRNPK	Yes
PTBP1	Yes
SFPQ	No
SRSF6	Yes

b

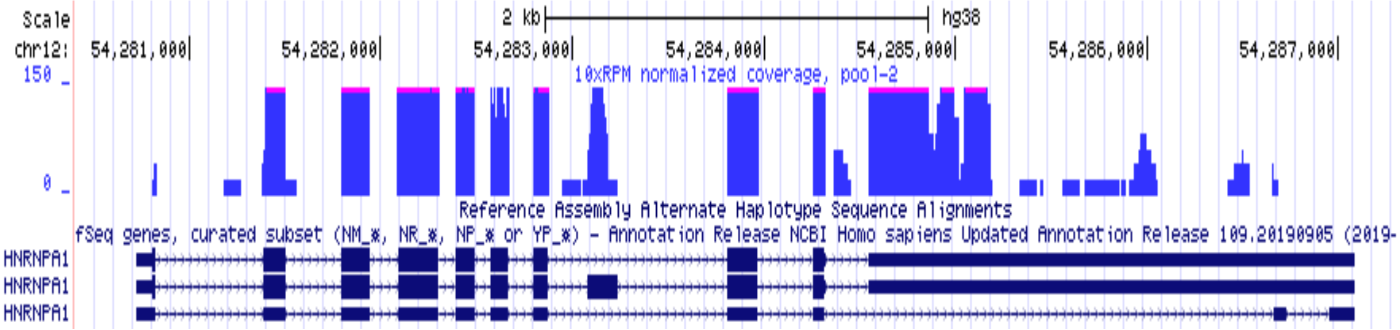

c

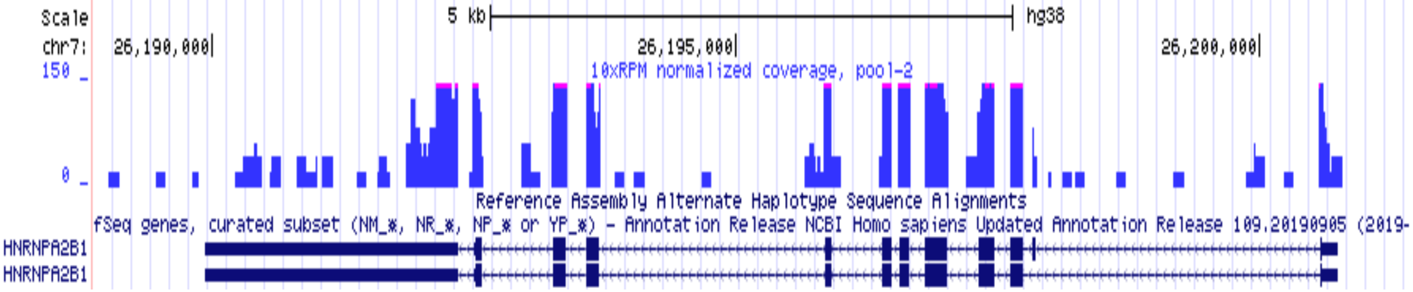

d

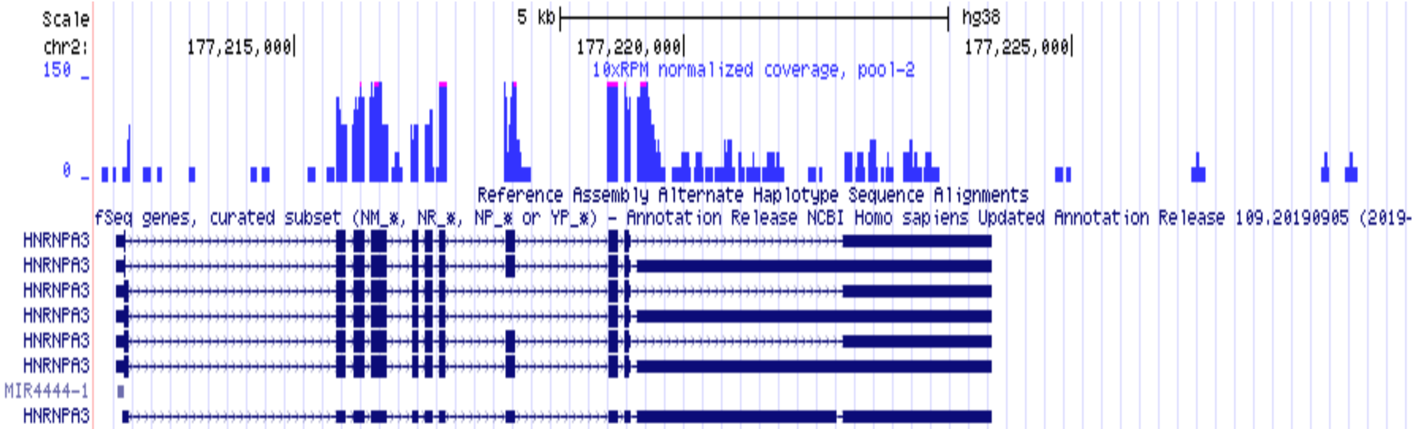

e

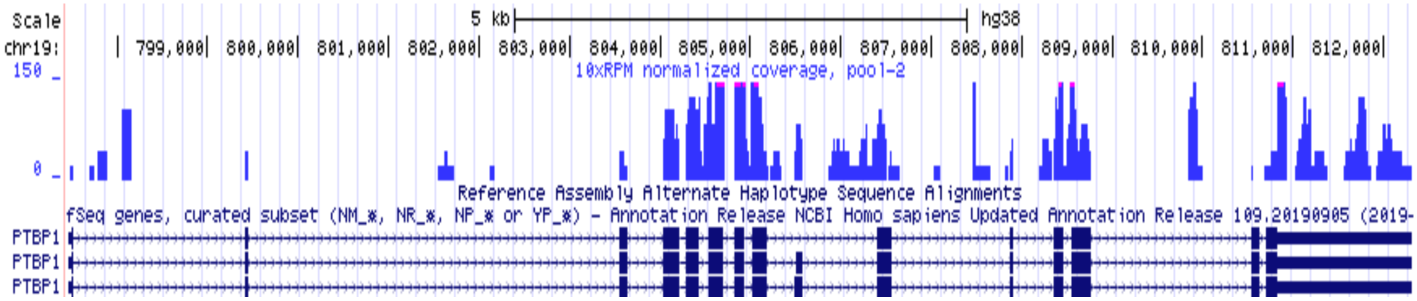

f

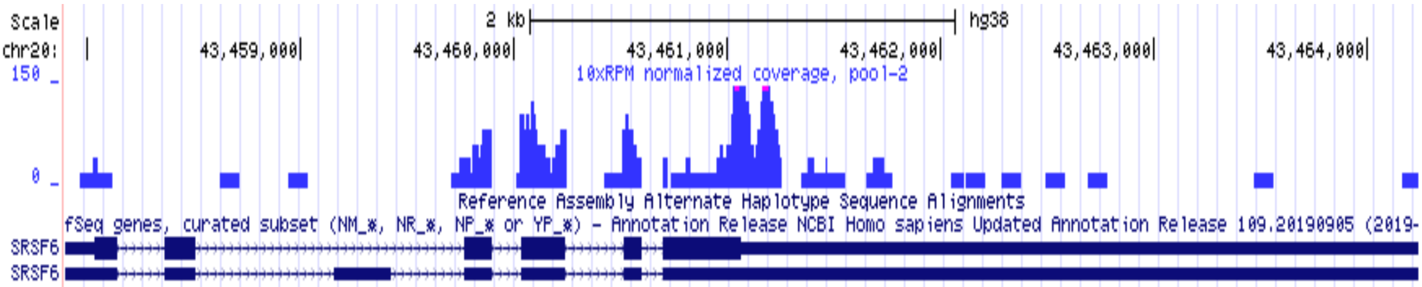

g

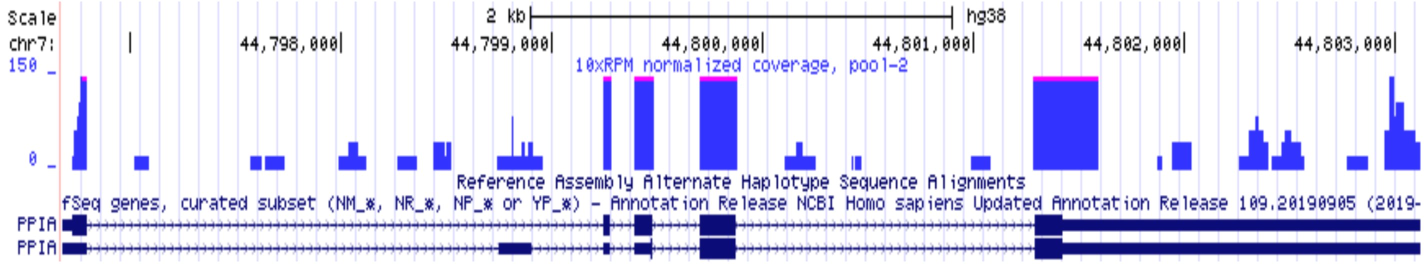

Supplementary Figure S1: a. To validate the presence of eight splicing factors in saliva, RNA was extracted from cell-free saliva from three different menstruating women with ages ranging from 20s to 50s and Real-time PCR was conducted. **b-g.** UCSC Genome Browser (reference genomeGRCh38/hg38). screen shot of RNA-seq data at the HNRNPA1 (**b**); HNRNPA2B1 (**c**); HNRNPA3 (**d**); PTBP1 (**e**); SRSF6 (**f**); and PPIA (**g**) locus genes.

Supplementary Fig. S2

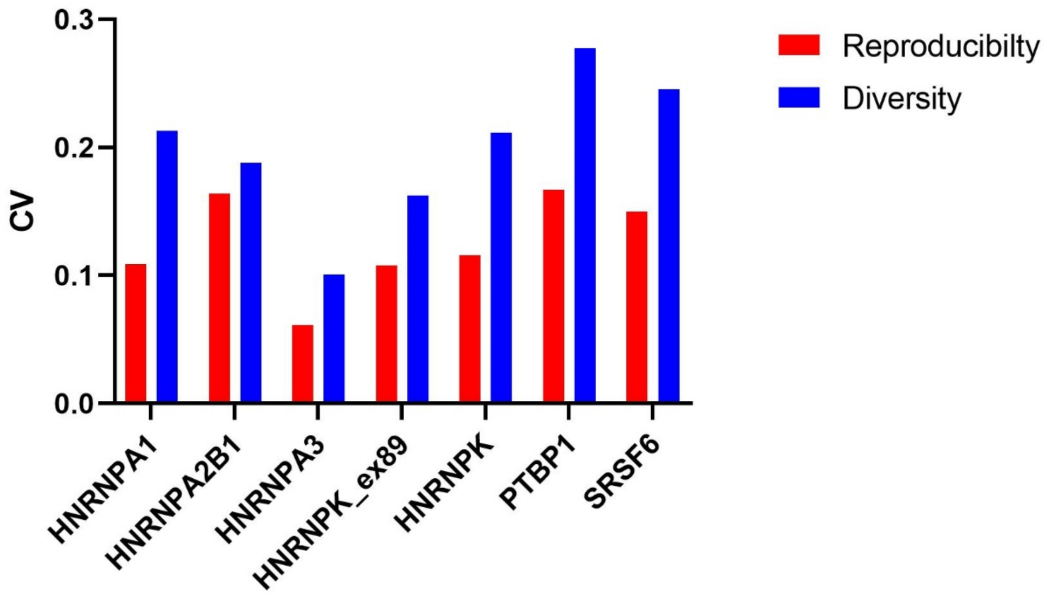

Supplementary Figure S2: For the reproducibility measurement, RNA was extracted from cell-free saliva from the same woman on different days. Diversity was measured by extraction of RNA from ten different menstruating women with ages ranging from 20s to 50s. Real-time PCR was conducted for HNRNPA1, HNRNPA2B1, HNRNPA3, HNRNPK, HNRNPK exon 8 inclusion, PTBP1 and SRSF6 and normalized to PPIA. Coefficient of variation (CV) statistical analysis was plotted.

Supplementary Fig. S3

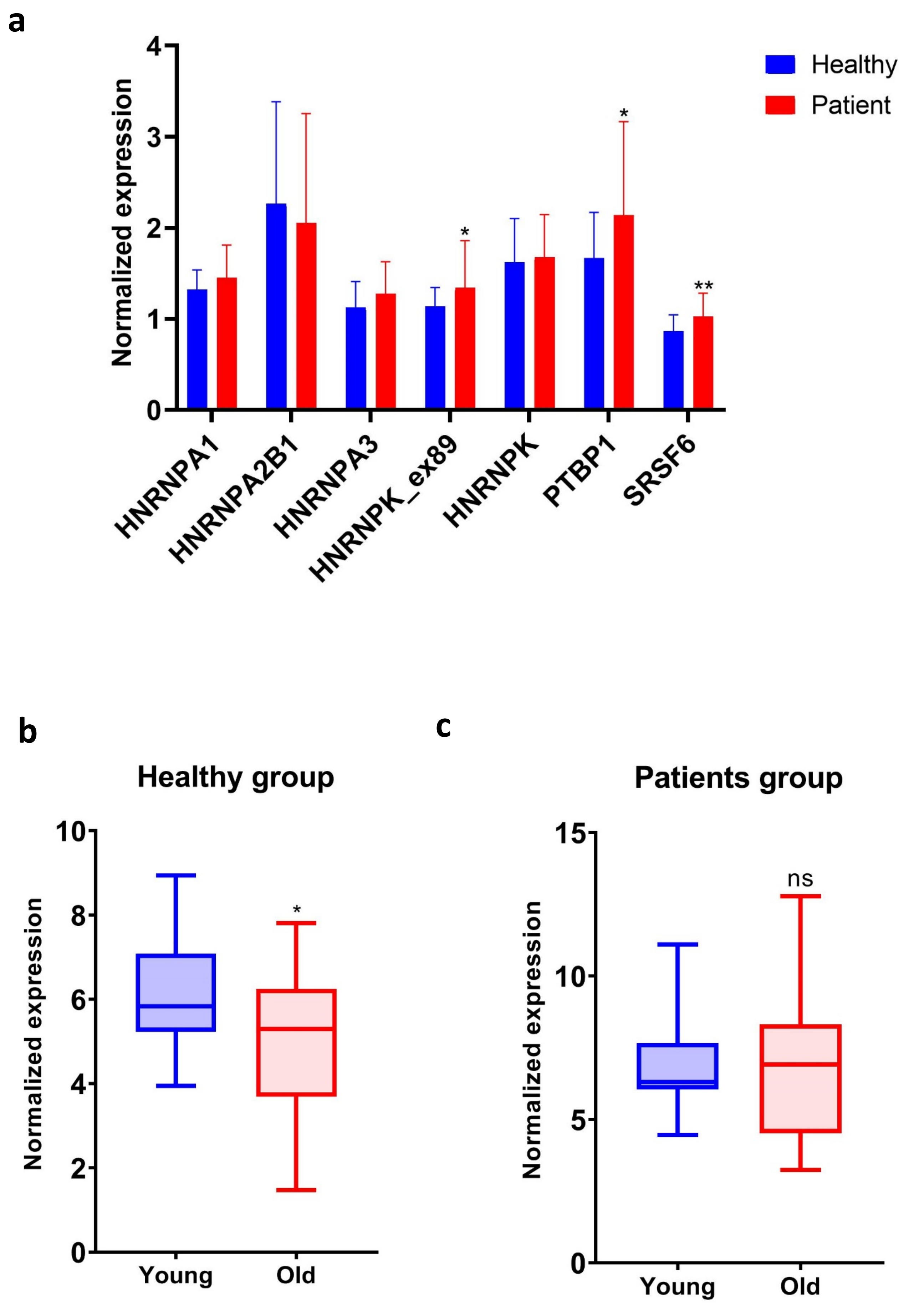

d

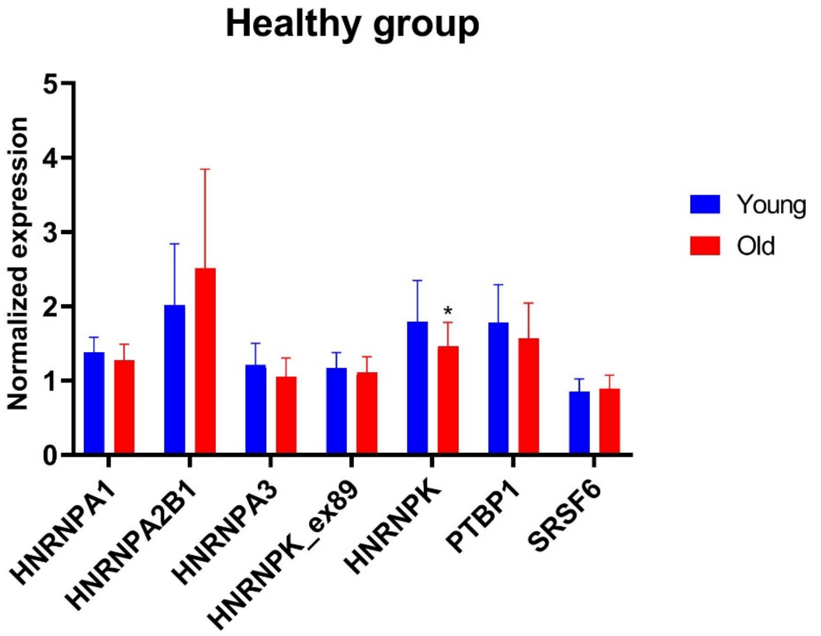

e

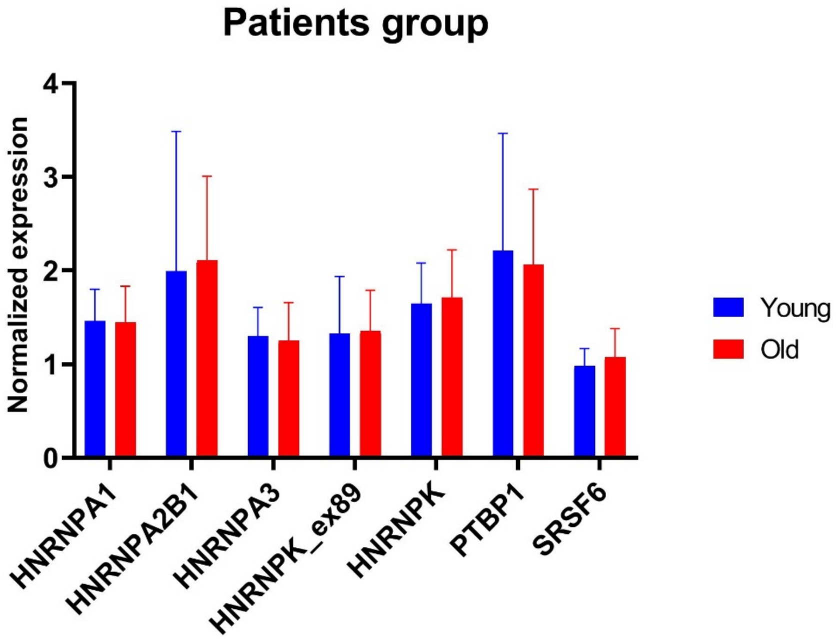

Supplementary Figure S3: a-e. RNA was extracted from cell-free saliva of 36 healthy and 31 breast cancer patients. Real-time PCR was conducted for HNRNPA1, HNRNPA2B1, HNRNPA3, HNRNPK, HNRNPK exon 8 inclusion, PTBP1 and SRSF6 and normalized to PPIA. Relative expression levels for each transcript were plotted **(a).** The data from 36 healthy subjects were pooled together and divided based on age to "young" and "old" groups. Relative expression levels were summed and plotted **(b).** The data from 31 breast cancer patients were pooled together and divided based on age to "young" and "old" groups. Relative expression levels were summed and plotted **(c).** The data from 36 healthy subjects were pooled together and divided based on age to "young" and "old" groups. Relative expression levels were summed and plotted for each splicing factor **(d).** The data from 31 breast cancer patients were pooled together and divided based on age to "young" and "old" groups. Relative expression levels were summed and plotted for each splicing factor **(e).**

Supplementary Table S1

**Oligo name**	**Sequence**
R_PPIA	CTGCTGTCTTTGGGACCTTGT
F_PPIA	GTCAACCCCACCGTGTTCTT
F-SRSF6-exon2	CCACGCACAAGCCATAGGCGAT
R-SRSF6-exon3	CGACCTGGAACGGGAGCGACT
f-HNRNPA1_total	GCTCACGGACTGTGTGGTAA
r-HNRNPA1_total	GGCCTTGCATTCATAGCTGC
f-HNRNPA2B1_total	CAGTTCTCACTACAGCGCCA
r-HNRNPA2B1_total	TTTCTCTCTCCATCGCGGAC
f-HNRNPA3_total	TGAAAGGGGGCAGTTTTGGT
r-HNRNPA3_total	ACTGTAGCCCTTTTCTGCTGT
f-HNRNPK_ex8-9	AATCTGATGCTGTGGAATGC
r-HNRNPK_ex8-9	ATTCCTCCTGCTAGACTCTG
f-HNRNPK_total	ACGCCCTGCAGAAGATATGG
r-HNRNPK_total	CCCCAGCATTCTTGCTCTGA
f-PTBP1_total	AGGGGAAAAACCAGGCCTTC
r-PTBP1_total	GGAGAGCTGTCGGTCTTCAG

Supplementary Table S2

CELF1
RBFOX2
HNRNPA1
HNRNPA2B1
HNRNPA3
HNRNPC
HNRNPD
HNRNPDL
HNRNPF
RBMX
HNRNPH1
PTBP1
HNRNPK
HNRNPL
HNRNPM
FUS
SYNCRIP
HNRNPU
KHSRP
MBNL1
SFPQ
KHDRBS1
SF3B1
SF1
SRSF1
SRSF3
SRSF6
YBX1
